# Is Younger Really Safer? A Qualitative Study of Perceived Risks and Benefits of Age-Disparate Relationships among Women in Cape Town, South Africa

**DOI:** 10.1371/journal.pone.0081748

**Published:** 2013-11-15

**Authors:** Roxanne Beauclair, Wim Delva

**Affiliations:** 1 The South African Department of Science and Technology/National Research Foundation (DST/NRF) Centre of Excellence in Epidemiological Modeling and Analysis (SACEMA), Stellenbosch University, Stellenbosch, South Africa; 2 International Centre for Reproductive Health, Ghent University, Gent, Belgium; Indiana University and Moi University, United States of America

## Abstract

Young women in age-asymmetric relationships may be at an elevated risk for acquisition of HIV, since relationships with older men are also correlated with other risk behaviors like less condom use. Qualitative studies have shown that women are motivated to participate in these relationships for money and emotional support. However, there is a paucity of research on women’s perceived risks of these relationships, particularly in South Africa. To this end, we conducted in-depth interviews with 23 women recruited from three urban communities in Cape Town. A thematic question guide was used to direct the interviews. Thematic content analysis was used to explore women’s perceived risks of age-disparate and non-age-disparate relationships, the benefits of dating older men, and risk perceptions that influence decisions around beginning or ending a relationship. A plurality of women thought that dating an older man does not bring any adverse consequences, although some thought that older men do not use condoms and may be involved in concurrent partnerships. Many women were less inclined to date same-age or younger men, because they were viewed as being disrespectful and abusive. This study points to the need for more awareness raising about the risks of age-disparate relationships. In addition to these initiatives, there is an urgent need to implement holistic approaches to relationship health, in order to curb intimate partner violence, improve gender equity and make non-age-disparate relationships more attractive.

## Introduction

Increasingly, epidemiological evidence is showing that age-asymmetric relationships—younger women engaging in sexual relationships with older men—may be at an elevated risk for sexually transmitted infections (STIs), including Human Immunodeficiency Virus (HIV) infection in sub-Saharan Africa [1–6]. The landmark study conducted by Gregson et al., demonstrates that for each year increase in the age difference between young women and their older male partners, the woman’s risk of also being HIV positive increases [2]. According to the 2008 South African National HIV Prevalence, Incidence, Behaviour and Communication Survey, the prevalence of infection is significantly higher among young women, with 21.1% of the 20-24 year-old women infected, compared to 5.1% of men infected in the same age category, [7]. The HIV prevalence in men peaks at 25.8% in the 30-34 year-old age group [7]. 

While the precise mechanism behind this gender discrepancy is incompletely understood, it is thought that a complex interplay of biological, socio-behavioral, and epidemiological factors is responsible for the observed differences in age-stratified HIV prevalence between men and women. Young women may be more vulnerable to HIV infection as a consequence of cervical ectopy (simple columnar epithelium is more susceptible to HIV and other STIs than squamous epithelium), an elevated inflammatory state of the female reproductive tract, and the HIV-susceptibility enhancing effect of injectable hormonal contraceptives [8], which are particularly popular among young women in many countries in sub-Saharan Africa [9]. The age-mixing pattern—how the sexual network connects individuals from different age groups—has also been identified as an important mediator of the spread of STIs [2]. At least two behavioral factors are thought to compound the effects of the biological factors and age-mixing pattern. First, evidence is accumulating to suggest that condom use is lower in men who engage in sexual relationships with younger women, compared to condom use in other men of the same age [1,10,11]. Second, older partners of young women have been known to sustain multiple concurrent partnerships (MCP) — overlapping sexual partnerships where sexual intercourse with one partner occurs between two acts of intercourse with another partner [2]. Concurrent relationships take place not only with casual partners, but also with main ‘long-term’ partners. The main partner of men engaging in concurrency is usually older than their casual partners [12], so these men may act as a bridging population, allowing HIV to spread indirectly from older age groups with a higher burden of HIV infections to younger age groups.

Qualitative research has shed light on the factors motivating young women to participate in age-asymmetric relationships in several sub-Saharan African countries. In Leclerc-Madlala’s review paper on age-asymmetric relationships, an age-disparate (AD) relationship is defined as one where there is an age difference of five or more years between partners, with the man being older than the woman in the relationship. An inter-generational/cross-generational relationship is a specific type of AD relationship, where the man is ten or more years older than the woman [13]. Often in literature on age mixing, the term ‘sugar daddies’ is used to refer to men in relationships with younger women, without regard to a specific minimum age difference [14]. It has been suggested that food insecurity and desperation may prompt a young woman to choose an intergenerational relationship [15]. Financial security and material objects that older men are purported to give are some of the more commonly cited incentives for age-asymmetric relationships [13,16–20]. Young women also specify psycho-social benefits such as feelings of love, increased self-esteem and self-confidence [13,17,21], as well as a raised status among peers [18] as reasons for engaging in age-asymmetric relationships. Some young women in Mozambique even enjoyed the freedom and independence that came from these relationships as there is often no expectation of permanency or prolonged affection [16]. Cultural prescriptions and adherence to traditional norms have also been cited as reasons for observing age-asymmetric relationships in sub-Saharan Africa [13].

Despite the mounting evidence about the risks of and motivations for age-asymmetric relationships, it is unclear how women perceive risks of engaging in AD relationships. Establishing how women perceive risks of engaging in AD relationships is necessary in order to inform public health interventions that attempt to limit these relationships and HIV transmission. To this end, we conducted in-depth, open-ended interviews in three different urban, Cape Town communities that are known to have a high prevalence of HIV. The study aimed to explore women’s perceptions of risky sexual behaviors and relationship dynamics. Here, we specifically report on their perceived risks of AD and non-age-disparate (non-AD) relationships, as well as women’s motivations for engaging in AD relationships. Risk perceptions that influence decisions around starting or breaking up sexual relationships were also explored.

## Materials and Methods

### Ethics Statement

Ethics approval was obtained from Stellenbosch University Health Research Ethics Committee. Written, informed consent was obtained from each participant and community consent was given by community advisory boards and ward councilors in each of the study communities. No compensation was given for any of the interviews.

### Setting

This qualitative study emerged from a previous cross-sectional sexual behavioral survey we conducted from June 2011 to February 2012 in three densely populated urban townships located on the periphery of Cape Town, South Africa. Upon conducting preliminary analysis of the survey data, additional questions began to arise about underlying reasons for risky behaviors including, but not limited to the observed age-mixing patterns. This prompted us to re-engage the three study communities, in March and April 2012, in order to explore relationship dynamics more thoroughly. The details of the antecedent study have been published elsewhere [22].

All three of the communities represent marginalized South African populations where the prevalence of HIV is high. According to the most recently available census data for the City of Cape Town, in all three of the communities more than 40% of the population is unemployed and less than 25% of the population has completed Grade 12 [23]. In two of the three study communities most residents live in informal shack dwellings [23]. In one of the communities participants were predominantly Black, and the other two communities had a combination of Black and Coloured participants. The term ‘Coloured’ is a South African moniker referring to the racially mixed descendants from Africa, Europe and slave populations of Asia. The three communities were chosen in order to gather a sample of Black and Coloured participants, since people identifying as either of these races often have limited access to financial, health, and educational services, and thus experience the brunt of the HIV epidemic in South Africa. 

### Population and Sample

We recruited 23 participants in total, in almost equal numbers from each of the three study communities, using a combination of maximum variation and snowball sampling methodologies. The researcher approached potential female participants, aged 18-65, who were located near the community town hall. Women belonging to a large range of ages were included in the sample in order to gather information about broader norms and beliefs held throughout society about sexual risk behaviors. The purpose of the study was explained to each woman approached, and then, if the woman agreed to participate, a venue and time for the interview was chosen. The number of women refusing to participate was not recorded. At the end of the interview, the women were asked to nominate other women they thought might be interested in participating in the interview. We approached the nominated women using the same method described above. Our sample size was determined by theoretical saturation. When no new concepts emerged we moved on to the next study community [24]. 

### Data Collection

The in-depth interviews were conducted by the first author, a female researcher who had postgraduate training in both anthropology and public health. The interviews were held in participants’ homes if privacy could be guaranteed. In a few of the cases the participants could not find a time to conduct the interviews at home when family members would not be around, so the researcher secured a safe room in the community hall where the interview was held in complete privacy. A thematic question guide was used to direct the interviews. It was composed of broad themes related to relationship dynamics. The themes were: ‘age-disparate relationships’, ‘starting and breaking up relationships’, ‘multiple concurrent partners’, ‘geographical location of partners’, ‘condom use’, ‘HIV behavioral interventions’, ‘alcohol use and sex’, and ‘partner turnover’. Throughout the interview, participants were allowed to change the direction and scope of the interview in new and meaningful ways. Additionally, the thematic question guide, itself, was not strictly adhered to from one interview to the next. The interviewer made changes in questioning based upon important and unanticipated topics that emerged from previous interviews. Before discussing AD relationships, we asked the participant if she knew what an AD relationship was and if she had heard of the term before. Upon hearing the participant’s answer, we provided her with the formal definition and allowed her to respond. The definition we used was: *an age-disparate relationship is one where the male partner is five or more years older than the female partner*. The researcher asked the questions in English and a research assistant was available to translate into isiXhosa or Afrikaans as necessary. Interviews lasted between 30 minutes and 90 minutes. To put the participants at ease, both researchers were female and neither the researcher nor the research assistant were from the study community, and thus, participants could be ensured that personal information would not be shared with the community. Field notes were made after the interview.

### Data Analysis

All interviews were digitally recorded, transcribed verbatim and subsequently translated to English. The data were analyzed using QSR International NVivo 9 software [25]. First, the first author coded all interviews according to the themes in the thematic question guide, developed by the second author. It quickly became apparent that ‘age-disparate relationships’ and ‘starting and breaking up relationships’ inspired the richest discussions and from them we gleaned the most novel insights into relationship dynamics. Then a thematic content analysis approach [26] was used to process information within those two themes. The first author read the transcripts line by line and did inductive coding. Themes, such as, ‘risks of non-AD relationships’ and ‘motivations of AD relationships’ emerged within these sections of the transcripts. Next, the first author examined text within these themes to see if any new lower-level themes materialized. Meanings and definitions were assigned to these lower-level themes. Both co-authors discussed the final coding and re-examined transcripts where necessary. The transcripts were not returned to participants for additional feedback primarily due to time constraints and difficulties in re-locating highly mobile participants in informal settlements. 

## Results

We interviewed 13 Black and 10 Coloured women, ranging from 20 to 59 years old. Seven of the participants openly discussed being in an AD relationship at the time of the interview. Several of the remaining 16 participants discussed past AD relationships they were involved in as well as ones they knew about at the time of the interview. Coding of these transcripts unearthed several themes related to motivations for participation in AD relationships, as well as perceived risks of AD and non-AD relationships. Finally, in our examination of participants’ reported reasons for beginning new relationships and terminating existing ones, we found connections to some of the reported risks of non-AD relationships.

### Motivations for AD relationships

Overwhelmingly, participants indicated that women were motivated to participate in AD relationships for financial or material benefits. One woman described various basic necessities that were easier to access with the help of an older male partner, “Sometimes let’s say you need money for, like, a train ticket, or like, (taxi) fare, or whatever, he can give you money, or if you don’t have … like shoes to wear, like, you know, he could buy you shoes as well” (Aged 40-44, Black). Other women indicated that the need for money, due to impoverishment and desperation, drove women to seek out relationships with older men. One woman stated, “And these young ladies they are also desperate, because the man is going to give the money to the lady, and the lady she’s going to pay (them) back” (Aged 25-29, Black). This woman alluded to the fact that some women may be explicitly engaging in transactional sex with older men to try ameliorate their hopeless situation. Another woman offered a similar explanation for her niece’s participation in AD relationships, “It comes from poverty, because I was giving (my niece) money, but … I wasn’t giving her enough money … And sometimes I don’t have money because my children are also in private school” (Aged 50-54, Black).

Many participants offered more cynical explanations for why women seek older men. Their articulated views often had an aura of condemnation and the young women were accused of ‘using’ older men for money:

It’s mostly younger women go for married men because sometimes you’ll find, like an example, let’s say, her neighbor is way older than her, and he’s married, and she doesn’t have everything like money or anything; life is not that good, so the neighbor, who is married, will offer those things to her. (Aged 25-29, Coloured)

There is some that think the older men, especially the Oupas (grandfathers), they take from them for the money and the house and the car. (Aged 45-49, Coloured)

It’s all about money because I don’t know of any young woman with an old man who doesn’t have money. I don’t know such a couple. I don’t know. If they have an old man … Does he have a taxi or a car? It’s all about his disability (grant) or his pension. (Aged 45-49, Coloured)

The participants also cited a few psychosocial benefits that result from AD relationships. Specifically, a few women thought that older men could tend to younger women’s needs to a greater extent than younger men. They said: “They know how to treat a woman” (Aged 40-44, Black), “An older man will take much better care of a younger woman” (Aged 55-59, Coloured), and “Because older men looks better after you than the younger people” (Aged 25-29, Coloured). The woman from the last quote elaborated by drawing on an example from someone she knew:

Almost like my friend has an older man who could have been her father. Almost like if she didn’t have a father figure throughout her childhood life, I assume that is also one of the reasons because maybe she didn’t get love and the attention of a father figure; that’s why she’s looking up to him and he gives her love and attention. (Aged 25-29, Coloured)

Echoing their sentiments, another woman said, “an older guy, he likes to spoil a woman when we … when you are young, like, he likes to spoil you a lot, and he’s got a … I think … he’s got much more love” (Aged 25-29, Black). Some women believed that an older man’s ability to care for a woman and provide love came from maturity that comes with age. As one woman said, “The older guy is more understanding because he’s got more life experience” (Aged 40-44, Coloured). Another participant said, “Mostly these younger guys, they want their freedom, they always want to go to the party or whatever, whereas you’d want him to stay at home with you, whereas an older guy, he is more mature and he’ll be able to be at home most of the time” (Aged 55-59, Coloured). 

Finally, two women expressed the idea that some younger women may choose to engage in AD relationships because of the prestige that is associated with dating older men who have had more opportunities and have acquired more wealth by virtue of their age.

Sometimes the woman will probably be in love with the person, regardless of what the person has. And then with some women, they date the man because he’s got his wealth, he’s got his money, he’s got his car, and he’s educated. (Aged 20-24, Black)

Let’s say I had an older guy, and you find that this guy … he’s got money, and he’s successful, OK? And most women they do it for those reasons. (Aged 20-24, Black)

The participants were very clear on the motivations young women have for participating in AD relationships, but questions remained about how young women were able to reconcile these motivations with the potential risks that AD relationships may pose for their health.

### Risks involved with AD relationships

A plurality of women, who shared their views about AD relationships, thought that no risks were involved with these relationships. Typical responses included: “From my experience, I don’t think there is any risks involved” (Aged 20-24, Black), “I don’t see any risk dating someone older” (Aged 20-24, Black), and “No. Like, there is no risk with being with an older man” (Aged 45-49, Coloured). Further probing of these particular women, about how AD relationships might specifically affect the spread of STIs, did not engender any more discussion on the topic. This is, perhaps, not surprising given that almost none of the women had heard of the term ‘age-disparate relationships’ prior to the interview, nor were they aware of any local health interventions that elucidated risks of AD relationships.

A small minority of women, however, did articulate some concerns with dating older men. They thought that older men refuse to wear condoms. One woman said, “They can’t eat a sweet with the wrapper on” (Aged 40-44, Black). Another stated:

(One of my friends) was telling me that ‘No, I will not use a condom with my boyfriend, because my boyfriend he doesn’t like to use a condom, and then he said that he will never use a condom at his age’, whereas he has never used a condom before, you see? ... So when I’m staying and thinking, I think ‘Ok, the old guys they say they’ve never used condoms before, and their grandfathers never used condoms’. So, that means all the older guys they don’t use condoms. (Aged 20-24, Black)

A few women explained that the risk to women actually came from the result of non-condom use. “Because sometimes he might not want to use a condom, and … a mistake could happen and you could, like, fall pregnant” (Aged 40-44, Black). Only two women were specifically concerned that the older men might have more STIs from not wanting to use condoms:

I’ll make an example. If I have an older man and I tell him to use a condom and he doesn’t want to use a condom, it already tells me something. Maybe this man is not right because he’s always supposed to. So … why don’t (he) want to condomize? (He) maybe can have a sick(ness), to make me sick. (Aged 45-49, Coloured)

The younger ones, they’ve grown up being taught about HIV and STIs, so the older group they didn’t have like a lot of … they weren’t aware of these diseases that we have today, so they are not really … they’re less likely to use condoms. (Aged 20-24, Black)

A small number of women were of the opinion that older men are more inclined to cheat, or in other words, have a concurrent relationship. As one woman stated, “You won’t know if that person has other multiple partners ... You’ll think that the person really loves you, but not knowing that that person is also busy with other people on the side” (Aged 20-24, Black). Another woman expressed that older men have access to a greater variety of age groups. “That older person have generations of other women” (Aged 25-29, Coloured). 

One of the older women in the sample explained that younger women are more naïve and thus not fully aware of all of the other women an older man may be sleeping with and potentially acquiring STIs from:

There are so many things that this man can successfully hide from (her) because of (his) advanced mindset. He can do it … he can have relationships behind her. This (young girl) is a toddler in front of him. She doesn’t know anything. As long as he’s giving her love, he can be involved with many other children that are younger than him, and this child can also die of infection. (Aged 50-54, Black)

One woman expressed sentiments about older men being more seasoned when it comes to having sexual relationships, and thus being more likely to have accumulated an STI:

Sometimes dating an older guy, you’ll find the guy’s more experienced. He’s been, like, in this relationship or dating scene for a long time. And then you’ll find that he’s had many multiple girlfriends, whatever, and then he gets infected by the virus, and then you start dating him, and then you don’t use a condom, then he infects you, and then that’s how it spreads. (Aged 20-24, Black)

### Perceived risks of non-AD relationships

Thematic content analysis results suggest that many women might be more concerned about risks associated with non-AD partnerships. Women were primarily concerned that these men might be more abusive and disrespectful to women. As three women remarked: “A guy that is younger, he tends to be more abusive and an older guy will not really lift his hand towards her” (Aged 55-59, Coloured), “Someone younger might be abusive” (Aged 20-24, Black), and “The young ones, they like being abusive and then the older men, he’s not like that” (Aged 30-34, Coloured). Another commented, “The younger guys…they’re disrespectful, they have no respect and they’re very abusive” (Aged 25-29, Black). This woman was particularly concerned about dating same-age men, because she thought they acted as though they had something to prove, “(If) you’re dating a younger guy, you will find that the reasons why most of these young guys, like, are abusive is because…that’s the way of claiming respect from you. The older guys they’re much nicer, and they don’t really, like, tend to be abusive” (Aged 25-29, Black). Another woman deemed same-age men to be more manipulative and emotionally abusive:

No please! I don’t like the youngsters. Yhu! ... because they like to play games … In the meantime they are going to … remind you that you’re an old woman. That is why I don’t like that stuff. I know that I’m old, and then now I’m involved with youngsters. And when the time goes on, he’s going to remind me every time that ‘… you know that you’re an old woman … you did date me, and you’re too old’. (Aged 25-29, Black)

Two women expressed concerns with younger men not providing support in one form or another. One woman described her own personal experience in a non-AD relationship: “I don’t trust him because he’s younger. I love him, he love me, that was fine … I’m older than him, I getting sick one of these days. He’s not going to look after me; he’s going to run, look for a wife his age. Then I will be a loser for the rest of my life” (Aged 45-49, Coloured). Another woman was worried that a non-AD partner would use her. “If you’re in a relationship with someone that’s closer to your age or younger than you, the guy would want you to buy him a lot of things, but in the relationship where you’re with an older guy, it will be more the guy who is able to spoil the woman instead of the woman spoiling the guy” (Aged 40-44, Coloured). 

Finally, one woman, who was in an AD relationship with a man nine years older than herself, indicated that men in non-AD relationships might be more likely to engage in multiple concurrent partnerships. She said, “Sometimes when you get a younger guy, they’re more into having multiple girlfriends. When you have an older guy, he’s more mature, ok?” (Aged 20-24, Black).

### Influence of risk perceptions on the dissolution of relationships

In the interviews women also discussed typical reasons for deciding to end relationships or seek new ones. The desire to avoid abuse and disrespect in their partnerships was a theme that surfaced in these discussions. When describing why she decided to date her current boyfriend, one woman said, “He is very caring and he would give me money, whereas my child’s father didn’t give me any money and he would just beat me up and just want to hurt the child” (Aged 55-59, Coloured). Other women articulated similar reasons for deciding to break up relationships based on prior experiences:

I won’t normally just end a relationship, but, because my ex-boyfriend was abusive, I just had to end that relationship. (Aged 25-29, Coloured)

If a guy he’s cheating on me, and…he’s not telling me the truth, and, ja, when the guy is abusing me, I quit. And … just … that’s all. (Aged 25-29, Black)

Ok the reason why I ended the relationship with the previous guy is because the guy wanted to kill my child. Wanted to stab the child, so I left him and I decided it’s just not right and then I met this [other] guy. (Aged 55-59, Coloured)

He was abusive and then if I’d go to work, like lets say I’d go to work the whole week and then Friday come back, I would get paid and he’d take my whole pay. So that’s why I ended that other relationship. (Aged 30-34, Coloured)

Maybe the person, like, drinks and then he becomes abusive. And secondly, like, if a person forcefully wants to have sex with you and you don’t want to. (Aged 40-44, Black)

Women also considered how respectful their potential partners would be in a relationship. To them, nominal levels of respect were usually required: they just wanted their partners to treat them and their children well. As one woman stated, “He’s got to have a very good heart and I’ve got two kids and he takes care of them and he’s very giving towards them” (Aged 30-34, Coloured). Another said, “I’m having a difficult time in terms of the person taking care of me. I’m worried about how he’ll take care of me” (Aged 55-59, Coloured). One woman’s requirements for initiating a relationship were that her suitor must treat her with courtesy and not be a criminal. “Respect is very important. And then, secondly, the person should have a job, and not go around robbing people” (Aged 25-29, Black). To many these may seem like low standards, but in these communities murders, gender-based violence and thefts are very common [27].

## Discussion

Our qualitative study about Cape Town women’s perspectives on relationship dynamics and sexual risk behavior revealed new and useful findings about risk perceptions of AD and non-AD relationships. In accord with other qualitative studies that have looked at motivations for participation in age-asymmetric relationships, our study also found that monetary support and material gain are primary drivers for this behavior [13,16–20]. Moreover, in concordance with recent literature from sub-Saharan Africa that describes how women exercise agency in choice of partner, as well as the duration of the partnership [13,16,18,20], we have also found that some of the participants described ‘other women’ actively seeking age-asymmetric relationships for psycho-social benefits like, affection, kind-treatment, and prestige. Additionally, some women showed evidence of agency in terminating relationships when they claimed to actively avoid abuse and disrespect. This suggests that many of the women in the study communities are not passive victims entering and staying in relationships out of desperation, but rather they are brokering respect from men whom they deem to be more kind.

Perhaps the most important finding is that a plurality of women did not perceive AD relationships to pose risks of any kind. Even those that perceived some risks did not necessarily make the connection between those risks and HIV/STI acquisition. The only other qualitative study, to our knowledge, that reported on perceived risks of dating older men, similarly found that both women and men were not concerned with STIs when engaging in these relationships because they judged their partners to be at low risk of having STIs [17]. 

Our study suggests that women might perceive older men to be a safer choice in comparison to non-AD partners. Women viewed relationships with the latter as particularly risky because they deemed non-AD partners more likely to be abusive. Indeed, women have much to be worried about as 42.3% of men, in a Cape Town-based study, admitted to having perpetrated intimate partner violence (IPV) the last 10 years [28]. Besides the immediate and undesirable threat of violence, qualitative research from South Africa has revealed that women who are victims of IPV believe that they are not in a position to demand condoms from their partners, or ask them to refrain from concurrent relationships [29]. Moreover, IPV has also been linked to other sexual risk indicators, such as meeting a sex partner at a bar/tavern, transactional sex, and recent STI diagnosis [30]. In South Africa, young men under the age of 25 who perpetrate IPV are also more likely to have HIV [31]. Likewise, women who experienced IPV in Rakai, Uganda were at increased risk of HIV infection [32]. It is unclear from our study if the women were aware of these additional risks associated with IPV. 

It remains equally uncertain whether or not women’s elevated concerns about IPV in relationships with younger men are warranted. In one study that looked at risk factors for committing IPV in South Africa, Gass et al. found that men’s age was not a determinant [33]. In studies that looked specifically at age difference as a risk factor for perpetrating or being a victim of IPV, results have been conflicting. Two studies, one conducted in rural South Africa among young women and the other in Denmark among men and women, found an association between having an older partner (>=3 years and >=5 years, respectively) and being a victim of IPV [34,35]. Two studies conducted in the U.S. found no significant association between age difference and female partners being a victim of IPV [36,37]. Finally, a study done in India found that an age difference of 5 years or less between spouses was a risk factor for the female partner being the victim of domestic violence [38]. Irrespective of which age groups truly inflict the most IPV, women’s perceptions and expectations are real and their decisions to dissolve relationships and seek new partners are, in part, motivated by their hope to avoid physical and emotional abuse, as we have pointed to in this study. Previous studies have pointed out that many women in sub-Saharan Africa choose to engage in AD relationships for different benefits that often outweigh the perceived risks of engaging in relationships with older men [13,39]. Our study questions whether AD relationships are perceived to be risky at all by a large fraction of women in poor communities with high HIV prevalence around Cape Town. Additionally, it begs the question whether the immediate threat of IPV has a stronger influence on relationship decisions among these women than the inconspicuous and more distant risk of HIV infection and other STIs.

This study conveys the need for a number of key interventions. First, educational and awareness-raising interventions that increase risk perception of AD relationships are currently inadequate and scarce [40,41]. Women need to have all the correct information about the specific types of risk that correspond with particular relationship types if they are to make more informed decisions regarding partner choice. Secondly, women’s concern with IPV signals the need for better services that help victims cope with clinical, psychological, social, and legal aspects of IPV. Some of the interventions needed for addressing IPV in these communities are: providing greater care for injuries, mental health, and unwanted pregnancies resulting from rape; delivering more peer support groups for coping with IPV; promoting development of safety plans with referrals to police and health services; and offering legal advice for obtaining protection orders [42]. These services have the potential to increase the social well-being of the affected individuals, as well as decrease anxiety, suicides and alcohol abuse among them [42]. Moreover, services such as these may start to make headway in addressing issues surrounding the substantive use of violence in these communities. These interventions, taken together, may make the less HIV risk-intensive relationships with same-age partners more attractive. 

In addition to these interventions, more epidemiological studies need to be conducted in South Africa to explore how risk of IPV and HIV is influenced by partner age differences. Looming questions remain about whether younger or same-age male partners do, in fact, perpetrate more IPV. Additionally, while there is some strong evidence to indicate that increasing age difference puts women at increased risk of being HIV positive, it is still unknown if there is a point at which age-difference stops being a risk factor. The epidemiological studies, to date, have examined this relationship using generalized linear regression models, whereby age difference is treated either as a continuous variable or as categorical variable with arbitrary categories (e.g. >=5 years older), which may mask which ranges of age differences convey the largest risk. Once enough evidence has been gathered, educational programs can attempt to address common misconceptions or truths about whom in their societies tend to be perpetrators of IPV, as well as provide women with information about which ‘older’ male partners may put them at the most risk of HIV. 

As with all qualitative studies, due to our relatively small, non-random sample, we cannot generalize our results to populations outside of urban and disadvantaged Cape Town communities. However, the use of maximum variation sampling procedures means that we were able to capture viewpoints from many types of women in the communities. Furthermore, we determined the sample size through theoretical saturation, which means that no new themes materialized by the time we decided to move on to the next community. By focusing on a limited number of communities only, we were able to gain a better understanding of what motivates women from these communities to choose AD relationships over the alternative, and thus gather context-specific information that will be more useful in developing content for interventions in the study communities. An additional limitation of this study is that some of the quotes from study participants reference other women’s behaviors, which may have limited the accuracy of the experiences they reported. Their opinions could represent hearsay or pluralistic ignorance. Even so, we do not think this was the case most of the time, as nearly one-third of the participants openly admitted to being in an AD relationship at the time of the study and many more admitted to past AD relationships. Moreover, most women did not perceive any grave risks associated with AD relationships or any stigma from participating in them, and therefore our study may have been less affected by social desirability bias. Finally, we are aware that our study is inclusive of age-mature women who may not be at an elevated risk of HIV acquisition through age-disparate relationships. The power dynamics between these women and men are enacted differently than those between younger women and older men. Despite this, we believe the inclusion of older female perspectives is necessary in order to gauge broader norms and beliefs held by society. Indeed, we found that many of the views were cross-generational and cross-racial in our sample.

Our study provides a unique contribution to qualitative literature on the topic of age-asymmetric relationships. To our knowledge, this is one of only two studies [17] to report on the perceived risks of AD and non-AD relationships. Additionally, through our investigation, we were able to generate a new hypothesis: that women’s preference for older men may be linked to their avoidance of IPV, thought to be perpetrated more frequently by younger or same-age partners.

## Conclusions

In conclusion, our study of women living in urban Cape Town communities with high HIV prevalence suggests that women may be choosing to engage in AD relationships in part because the alternative—relationships with same-age or younger men—poses the more immediate and severe threat of IPV. In addition to initiatives that raise more awareness about the risks of AD relationships, there is an urgent need to implement holistic approaches to relationship health, in order to curb IPV, improve gender equity and make relationships with peers more attractive.

## References

[1] Bankole A, Ahmed FH, Neema S, Ouedraogo C, Konyani S (2007) Knowledge of correct condom use and consistency of use among adolescents in four countries in Sub-Saharan Africa. Afr J Reprod Health 11: 197-220. 18458741 PMC2367135

[2] Gregson S, Nyamukapa CA, Garnett GP, Mason PR, Zhuwau T et al. (2002) Sexual mixing patterns and sex-differentials in teenage exposure to HIV infection in rural Zimbabwe. Lancet 359: 1896-1903. 12057552 10.1016/S0140-6736(02)08780-9

[3] Kelly RJ, Gray RH, Sewankambo NK, Serwadda D, Wabwire-Mangen F et al. (2003) Age differences in sexual partners and risk of HIV-1 infection in rural Uganda. J Acquir Immune Defic Syndr 32: 446-451. 12640205 10.1097/00126334-200304010-00016

[4] Sa Z, Larsen U (2008) Gender inequality increases women's risk of hiv infection in Moshi, Tanzania. J Biosoc Sci 40: 505-525. 18088449 10.1017/S002193200700257X

[5] Shisana O, Rehle T, Simbayi C, Parker W, Zuma K et al. (2005) South African national HIV prevalence, HIV incidence, behavioural and communication survey, 2005. Cape Town: Human Sciences Research Council.

[6] Beauclair R, Kassanjee R, Temmerman M, Welte A, Delva W (2012) Age-disparate relationships and implications for STI transmission among young adults in Cape Town, South Africa. Eur J Contracept Reprod Health Care 17: 30-39. doi:10.3109/13625187.2011.644841. 22239263

[7] Shisana O, Rehle T, Simbayi L, Zuma K, Jooste S et al. (2009) South African national HIV prevalence, incidence, behaviour and communication survey 2008: A turning tide among teenagers? Cape Town: Human Sciences Research Council.

[8] Yi TJ, Shannon B, Prodger J, McKinnon L, Kaul R (2013) Genital immunology and HIV susceptibility in young women. Am J Reprod Immunol 69 Suppl 1: 74-79. doi:10.1111/aji.12035. 23157424

[9] Butler AR, Smith JA, Polis CB, Gregson S, Stanton D et al. (2013) Modelling the global competing risks of a potential interaction between injectable hormonal contraception and HIV risk. AIDS 27: 105-113. doi:10.1097/QAD.0000000000000040. 23014519 PMC4862571

[10] Luke N (2005) Confronting the 'sugar daddy' stereotype: age and economic asymmetries and risky sexual behavior in urban Kenya. Int Fam Plan Perspect 31: 6-14. doi:10.1363/3100605. 15888404

[11] Langeni T (2007) Contextual factors associated with treatment-seeking and high-risk sexual behavior in Botswana among men with symptoms of sexually transmitted infections. Afr J AIDS Res 6: 261-269. doi:10.2989/16085900709490422. 25866172

[12] Chopra M, Townsend L, Johnston L, Mathews C, Tomlinson M et al. (2009) Estimating HIV prevalence and risk behaviors among high-risk heterosexual men with multiple sex partners: use of respondent-driven sampling. J Acquir Immune Defic Syndr 51: 72-77. doi:10.1097/QAI.0b013e31819907de. PubMed: 19282783. 19282783

[13] Leclerc-Madlala S (2008) Age-disparate and intergenerational sex in southern Africa: the dynamics of hypervulnerability. AIDS 22 Suppl 4: S17-S25. doi:10.1097/01.aids.0000343761.77702.04. PubMed: 19033752. 19033752

[14] Kuate-Defo B (2004) Young People's Relationships with Sugar Daddies and Sugar Mummies: What Do We Know and What Do We Need to Know? Afr J Reprod Health 8: 13-27. doi:10.2307/3583175. PubMed: 15623116. 15623116

[15] Weiser SD, Leiter K, Bangsberg DR, Butler LM, Percy-de Korte F et al. (2007) Food insufficiency is associated with high-risk sexual behavior among women in Botswana and Swaziland. PLOS Med 4: 1589-1597; discussion: 17958460. 17958460 10.1371/journal.pmed.0040260 PMC2039764

[16] Hawkins K, Price N, Mussa F (2012) Milking the cow: Young women's construction of identity and risk in age-disparate transactional sexual relationships in Maputo, Mozambique. Global Public Health: An International Journal for Research, Policy and Practice 4: 169-182. 10.1080/17441690701589813 19333807

[17] Longfield K, Glick A, Waithaka M, Berman J (2004) Relationships between older men and younger women: Implications for STIs/HIV in Kenya. Stud Fam Plan 35: 125–34. PubMed: 15260214. 10.1111/j.1728-4465.2004.00014.x 15260214

[18] Luke N (2003) Age and economic asymmetries in the sexual relationships of adolescent girls in sub-Saharan Africa. Stud Fam Plann 34: 67-86. doi:10.1111/j.1728-4465.2003.00067.x. PubMed: 12889340. 12889340

[19] Moore AM, Biddlecom AE, Zulu EM (2007) Prevalence and meanings of exchange of money or gifts for sex in unmarried adolescent sexual relationships in sub-Saharan Africa. Afr J Reprod Health 11: 44-61. doi:10.2307/25549731. PubMed: 18458736. 18458736 PMC2367111

[20] Groes-Green C (2013) "To put men in a bottle": Eroticism, kinship, female power, and transactional sex in Maputo, Mozambique. Am Ethnologist 40: 102-117. doi:10.1111/amet.12008.

[21] Nkosana J, Rosenthal D (2007) The dynamics of intergenerational sexual relationships: the experience of schoolgirls in Botswana. Sex Health 4: 181-187. doi:10.1071/SH06070. PubMed: 17931531. 17931531

[22] Delva W, Beauclair R, Welte A, Vansteelandt S, Hens N et al. (2011) Age-disparity, sexual connectedness and HIV infection in disadvantaged communities around Cape Town, South Africa: a study protocol. BMC Public Health 11: 616. doi:10.1186/1471-2458-11-616. PubMed: 21810237. 21810237 PMC3161892

[23] Town CoC (2001) City of Cape Town-Census 2001. Cape Town: Strategic Development Information.

[24] Marshall MN (1996) Sampling for qualitative research. Fam Practice 13: 522-525. doi:10.1093/fampra/13.6.522. PubMed: 9023528. 9023528

[25] NVivo qualitative data analysis software: QSR International Pty Ltd.

[26] Green J, Thorogood N (2004) Qualitative Methods for Health Research. London: SAGE Publications Ltd.

[27] Mayosi BM, Lawn JE, van Niekerk A, Bradshaw D, Abdool Karim SS et al. (2012) Health in South Africa: changes and challenges since 2009. Lancet 380: 2029-2043. doi:10.1016/S0140-6736(12)61814-5. PubMed: 23201214. 23201214

[28] Abrahams N, Jewkes R, Laubscher R, Hoffman M (2006) Intimate partner violence: prevalence and risk factors for men in Cape Town, South Africa. Violence Vict 21: 247-264. doi:10.1891/vivi.21.2.247. PubMed: 16642742. 16642742

[29] Fox AM, Jackson SS, Hansen NB, Gasa N, Crewe M et al. (2007) In their own voices: A qualitative study of women's risk for intimate partner violence and HIV in South Africa. Violence Against Women 13: 583–602. doi:10.1177/1077801207299209. PubMed: 17515407. 17515407

[30] Pitpitan EV, Kalichman SC, Eaton LA, Cain D, Sikkema KJ et al. (2012) Gender-based violence, alcohol use, and sexual risk among female patrons of drinking venues in Cape Town, South Africa. J Behav Med, 36: 295–304. PubMed: 22526526. 22526526 10.1007/s10865-012-9423-3 PMC3646073

[31] Jewkes R, Sikweyiya Y, Morrell R, Dunkle K (2011) The relationship between intimate partner violence, rape and HIV amongst South African men: a cross-sectional study. PLOS ONE 6: e24256. doi:10.1371/journal.pone.0024256. PubMed: 21935392. 21935392 PMC3173408

[32] Kouyoumdjian FG, Calzavara LM, Bondy SJ, O'Campo P, Serwadda D et al. (2013) Intimate partner violence is associated with incident HIV infection in women in Uganda. AIDS 27: 1331-1338. PubMed: 23925380. 23925380 10.1097/QAD.0b013e32835fd851

[33] Gass JD, Stein DJ, Williams DR, Seedat S (2011) Gender differences in risk for intimate partner violence among South African adults. J Interpers Violence 26: 2764-2789. doi:10.1177/0886260510390960. PubMed: 21156693. 21156693 PMC3281490

[34] Jewkes R, Dunkle K, Nduna M, Levin J, Jama N et al. (2006) Factors associated with HIV sero-positivity in young, rural South African men. Int J Epidemiol 35: 1455-1460. doi:10.1093/ije/dyl217. PubMed: 17030525. 17030525

[35] Faergemann C, Lauritsen JM, Brink O, Mortensen PB (2010) Do repeat victims of interpersonal violence have different demographic and socioeconomic characters from non-repeat victims of interpersonal violence and the general population? A population-based case-control study. Scand J Public Health 38: 524-532. doi:10.1177/1403494810370234. PubMed: 20484309. 20484309

[36] Roberts TA, Auinger P, Klein JD (2006) Predictors of partner abuse in a nationally representative sample of adolescents involved in heterosexual dating relationships. Violence Vict 21: 81-89. doi:10.1891/0886-6708.21.1.81. PubMed: 16494134. 16494134

[37] Volpe EM, Hardie TL, Cerulli C, Sommers MS, Morrison-Beedy D (2013) What's age got to do with it? Partner age difference, power, intimate partner violence, and sexual risk in urban adolescents. J Interpers Violence 28: 2068-2087. doi:10.1177/0886260512471082. PubMed: 23345572. 23345572 PMC3706999

[38] Pandey GK, Dutt D, Banerjee B (2009) Partner and relationship factors in domestic violence: perspectives of women from a slum in Calcutta, India. J Interpers Violence 24: 1175-1191. doi:10.1177/0886260508322186. PubMed: 18840848. 18840848

[39] Leclerc-Madlala S (2004) Transactional Modernity, Sex and the Pursuit of Modernity. Soc Dynam 2: 1-21.

[40] Kelly K, Mkhwanazi N, Nkhwashu N, Rapiti R, Mashale R (2012) HIV prevention situation analysis in KwaZulu-Natal, Mpumalanga and Gauteng provinces, South Africa. USAID Sexual HIV Prevention Programme in South Africa (SHIPP).

[41] Hope R (2007) Addressing Cross-Generational Sex: A desk review of research and programs. Population Reference Bureau.

[42] Joyner K, Mash RJ (2011) The value of intervening for intimate partner violence in South African primary care: project evaluation. BMJ Open 1: e000254. doi:10.1136/bmjopen-2011-000254. PubMed: 22146888. PMC3236818 22146888

